# Clinical predictive value of the age, creatinine, and ejection fraction score in patients in acute type A aortic dissection after total arch replacement

**DOI:** 10.1038/s41598-024-58608-4

**Published:** 2024-05-11

**Authors:** Xin-fan Lin, Lin-feng Xie, Zhao-feng Zhang, Qing-song Wu, Zhi-huang Qiu, Liang-wan Chen

**Affiliations:** 1 Department of Cardiovascular Surgery, Fujian Medical University Union Hospital, Xinquan Road 29, Fuzhou, 350001 Fujian People’s Republic of China; 2 Key Laboratory of Cardio-Thoracic Surgery (Fujian Medical University), Fujian Province University, Fuzhou, Fujian People’s Republic of China; 3 Fujian Provincial Center for Cardiovascular Medicine, Fuzhou, Fujian People’s Republic of China

**Keywords:** Biomarkers, Cardiology, Risk factors

## Abstract

The age, creatinine, and ejection fraction (ACEF) score has been accepted as a predictor of poor outcome in elective operations. This study aimed to investigate the predictive value of ACEF score in acute type A aortic dissection (AAAD) patients after total arch replacement. A total of 227 AAAD patients from July 2021 and June 2022 were enrolled and divided into Tertiles 1 (ACEF ≤ 0.73), Tertiles 2 (0.73 < ACEF ≤ 0.95), and Tertiles 3 (ACEF > 0.95). Using inverse probability processing weighting (IPTW) to balance the baseline characteristics and compare the outcomes. Cox logistic regression was used to further evaluate the survival prediction ability of ACEF score. The in-hospital mortality was 9.8%. After IPTW, in the baseline characteristics reached an equilibrium, a higher ACEF score before operation still associated with higher in-hospital mortality. After 1 year follow-up, 184 patients (90.6%) survival. Multivariable analysis revealed that ACEF score (adjusted hazard ratio  1.68; 95% confidence interval 1.34–4.91; *p* = 0.036) and binary ACEF score (adjusted HR 2.26; 95% CI 1.82–6.20; *p* < 0.001) was independently associated with 1-year survival. In addition, net reclassification improvement (NRI) and integrated differentiation improvement (IDI) verified that the ACEF score and binary ACEF score is an accurate predictive tool in clinical settings. In conclusions, ACEF score could be considered as a useful tool to risk stratification in patients with AAAD before operation in daily clinical work.

## Introduction

The acute Stanford type A aortic dissection (AAAD) is a devastating condition with poor prognosis^1^. Technological advances in cardiac surgery have led to personalized treatment options for each situation^2^. Therefore, preoperative quick screening high-risk patients is of particular importance to tailor and optimize perioperative strategy. In the last few decades, several risk scoring systems for stratification of patients undergoing cardiac surgery have been proposed. Of those, the European System for Cardiac Operative Risk Evaluation II (EuroSCORE II)^3^, and the German Registry for Acute Aortic Dissection Type A (GERAADA) score^4^ are the best known. The GERAADA score, which is specifically designed for predicting 30-day mortality in patients undergoing surgery for AAAD, contains a majority of the risk factors implemented in the EuroSCORE II^5^. The age, creatinine, and ejection fraction (ACEF) score is a novel and simple risk assessment tool which includes only three variables, initially developed in the prediction of mortality in patients undergoing elective cardiac operations^6^. And further independent validated in several types of cardiovascular surgery, such as valve surgery and ventricular reconstruction^7–9^. However, data on the ACEF score in patients with AAAD are scarce. Consequently, the present study was conducted to assess the predictive value of the ACEF score in patients with AAAD after total arch replacement.

## Methods

### Patient population

**Figure 1:**
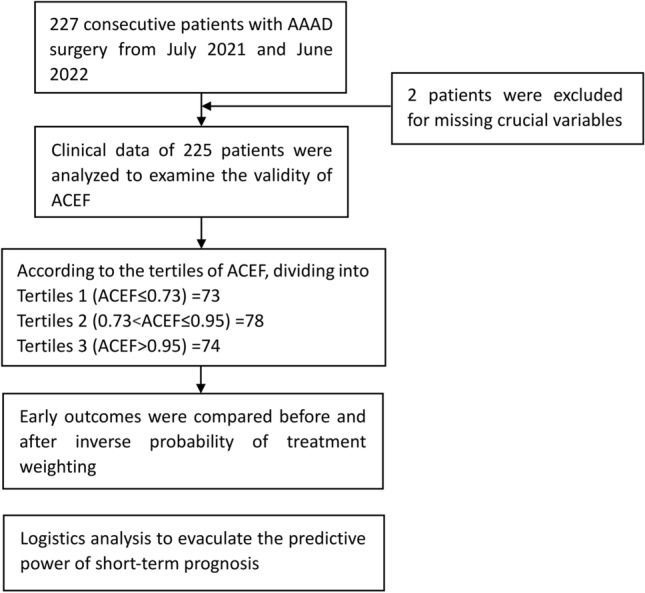
The flowchart of patient selection and analysis.

This was a retrospective, single-center observational study of patients with AAAD underwent total arch replacement in our institution between July 2021 and June 2022. The diagnosis was made according to computed tomography angiography (CTA) and echocardiography. All the patients met the indications for total arch replacement which was described in our previous study^10^. The exclusion criteria were as follows: (1) less than 18 years; (2) sub-acute and chronic AD (the diagnosis was made according to onset time and imaging^11^); (3) absence of crucial clinical data (required to calculate the ACEF I or II were missing). Figure 1 provides an overview of the study procedures.

The present study was following the guidelines of the Declaration of Helsinki and was approved by the Medical Ethics Committee of Fujian Medical University Union Hospital. This retrospective study had the approval from the ethics committee, with the need for informed consent from patients was waived.

### Data collection and calculation

The patients were divided into three groups separated by the 33rd and 67th percentiles (tertiles) of ACEF score. Clinical data were collected from electronic medical records by one researcher and checked by another researcher randomly. Preoperative imaging examinations and laboratory tests were performed at emergency admission before any treatment. All in-hospital survival patients were followed up via outpatient clinic visits or telephone interviews after discharge. The follow-up data, including follow-up time and status, was collected.

As suggested by Ranucci et al., the ACEF score was calculated as follows: age (years) / left ventricular ejection fraction (LVEF) (%) + 1 (if serum creatinine > 2 mg/dl)^6^.

the ACEF II score was calculated as follows: age (years) / LVEF (%) + 2 (if serum creatinine > 2 mg/dl) + 3 (if emergency surgery) + 0.2 × (hematocrit points below 36%)^12^.

### Endpoints

For the purposes of the present study, the primary endpoint was all-cause mortality. The secondary outcome was severe postoperative complications, defined by The International Aortic Arch Surgery Study Group (IAASSG)^13^, requiring intervention under regional or general anesthesia or requiring new ICU admission or ongoing ICU management for > 7 d or hospitalization for > 30 d, or causing secondary organ failure^14^.

### Statistical analysis

The statistical analysis was conducted in R software version 4.3.2. Continuous variables were reported as mean ± SD or median and quartile range, and compared using analysis of variance or Mann–Whitney test. Categorical variables were expressed as percentages and compared by chi-square analysis. To ensure balance in baseline characteristic, inverse probability of treatment weighting (IPTW) was utilized. Compared to propensity score matching (PSM), IPTW can minimize the influence of confounding variables while maximizing the amount of available information. The sample size formed after IPTW is based on the weight and the pseudo sample size generated by the actual sample^15^. Discrimination performance of ACEF, ACEF II score in predicting mortality was evaluated by receiver-operating characteristic (ROC) analysis. Univariate Cox proportional hazards model analysis was conducted to determine the independent predictors of 1-year rates of mortality. The variables whose *p* < 0.10, except the variables of ACEF score, were included in a multivariate model for further analysis. The best cutoff value of ACEF value was identified according to the Youden index. To determine whether the model improved after rounding of continuous ACEF for categorization, Harrell’s C-index, time dependent net reclassification improvement (NRI) and time dependent integrated discrimination improvement (IDI) were calculated. Furthermore, time-dependent (3, 6, 12 month) ROC analysis was conducted. A value of *p* < 0.05 was considered significant.

### Ethics approval

The present study was following the guidelines of the Declaration of Helsinki and was approved by the Medical Ethics Committee of Fujian Medical University Union Hospital.

### Patient consent

This retrospective study had the approval from the ethics committee of Fujian Medical University Union Hospital, with the need for informed consent from patients was waived.

## Results

### Baseline characteristics

**Figure 2:**
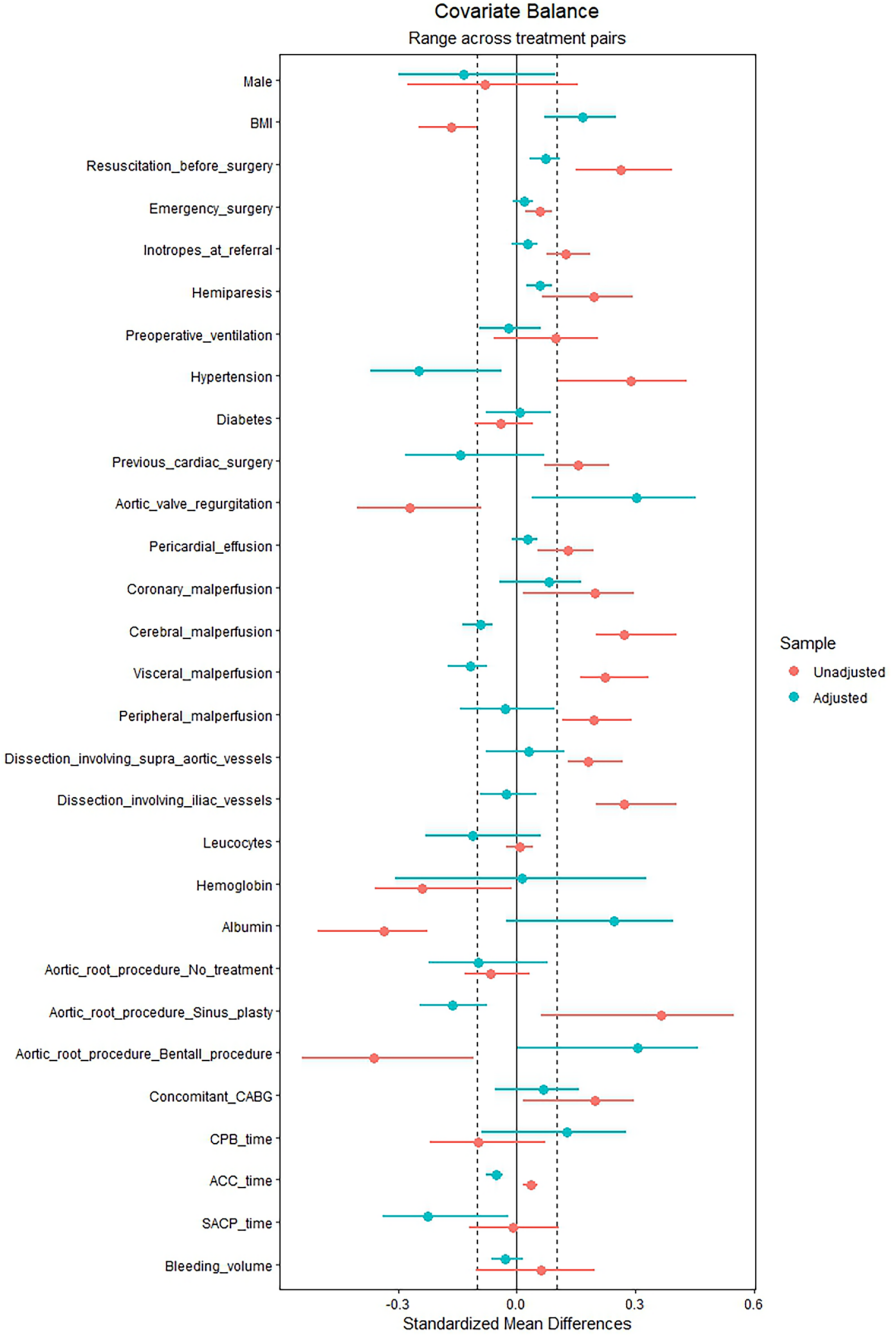
A Love plot was used to assessed standardized mean differences before adjusting and after adjusting.

We totally enrolled 227 patients who had undergone total arch replacement. 2 patients were excluded for missing key data. After exclusions, a total of 225 AAAD patients for further analysis. The median age was 51.24 ± 11.43 years, with 131 (58.22%) males and 94 (41.78%) females. Patients were classified into three groups according to the tertiles of ACEF score: Tertiles 1 (ACEF ≤ 0.73, n = 73), Tertiles 2 (0.73 < ACEF ≤ 0.95, n = 78) and Tertiles 3 (ACEF > 0.95, n = 74). Obviously, significant differences were observed among tertiles in age, serum creatinine and LVEF. In addition, patients with a high ACEF score had a higher proportion of hypertension (*p* = 0.027) and more likely to receive a simpler root procedure (*p* = 0.003). In order to make all the data comparable, the IPTW was conducted to match the baseline and surgical variables aside from age, serum creatinine, LVEF. After matching, no else significant differences were detected. Table 1 Summarizes the baseline characteristics comparison between groups before and after IPTW (Fig. 2). The standardized mean differences were assessed graphically by using a Love plot. All covariates that had a standardized mean difference of < 0.25 were determined to be balanced.

**Table 1:** Baseline characteristics and clinical characteristics according to ACEF score groups before matching and after IPTW.

Parameters	ACEF score tertiles									
	Before matching					After IPTW				
	Overall	≤ 0.73	0.73–0.95	> 0.95	*p*	Overall	≤ 0.73	0.73–0.95	> 0.95	*p*
n	225	73	78	74	NA	239.97	70.57	70.81	98.6	NA
Demographical and clinical variables										
Age (years)	51.24 ± 11.43	39.62 ± 7.45	53.99 ± 5.60	59.80 ± 9.77	< 0.001	53.11 ± 12.09	39.77 ± 8.79	53.87 ± 6.01	62.11 ± 8.12	< 0.001
Male	131 (58.22)	43 (58.90)	40 (51.28)	48 (64.86)	0.234	78.82 (32.84)	25.04 (35.48)	28.51 (40.26)	25.27 (25.63)	0.345
BMI (kg/m^2^)	25.19 ± 3.15	25.56 ± 3.65	25.24 ± 2.94	24.77 ± 2.80	0.316	25.18 ± 2.93	24.69 ± 3.57	25.26 ± 2.92	25.48 ± 2.36	0.533
Resuscitation before surgery	7 (3.11)	0 (0.00)	2 (2.56)	5 (6.76)	0.058	2.78 (1.16)	0.00 (0.00)	0.94 (1.32)	1.84 (1.87)	0.339
Emergency surgery	198 (88.00)	63 (86.30)	69 (88.46)	66 (89.19)	0.855	212.68 (88.63)	62.34 (88.34)	62.30 (87.97)	88.05 (89.31)	0.968
Inotropes at referral	11 (4.89)	2 (2.74)	4 (5.13)	5 (6.76)	0.525	7.20 (3.00)	1.62 (2.29)	2.44 (3.44)	3.14 (3.19)	0.901
Hemiparesis	8 (3.56)	1 (1.37)	2 (2.56)	5 (6.76)	0.178	4.22 (1.76)	0.53 (0.75)	1.36 (1.93)	2.33 (2.37)	0.606
Preoperative ventilation	8 (3.56)	1 (1.37)	4 (5.13)	3 (4.05)	0.442	5.72 (2.38)	1.62 (2.30)	2.42 (3.42)	1.67 (1.69)	0.757
Hypertension	172 (76.44)	48 (65.75)	62 (79.49)	62 (83.78)	0.027	168.98 (70.42)	54.53 (77.27)	53.58 (75.66)	60.88 (61.75)	0.427
Diabetes	12 (5.33)	4 (5.48)	5 (6.41)	3 (4.05)	0.810	9.82 (4.09)	2.42 (3.43)	3.81 (5.38)	3.59 (3.64)	0.805
Previous cardiac surgery	7 (3.11)	1 (1.37)	2 (2.56)	4 (5.41)	0.349	8.15 (3.39)	4.49 (6.36)	1.04 (1.47)	2.61 (2.65)	0.391
LVEF (%)	63.65 ± 7.01	66.08 ± 5.31	64.92 ± 5.57	59.91 ± 8.28	< 0.001	64.10 ± 6.67	66.27 ± 4.98	64.75 ± 5.75	62.08 ± 7.73	0.057
Aortic valve regurgitation	65 (28.89)	29 (39.73)	20 (25.64)	18 (24.32)	0.076	80.52 (33.55)	17.46 (24.74)	18.75 (26.48)	44.31 (44.94)	0.236
Pericardial effusion	10 (4.44)	2 (2.74)	3 (3.85)	5 (6.76)	0.473	5.33 (2.22)	1.10 (1.56)	1.88 (2.65)	2.35 (2.39)	0.846
End-organ malperfusion					NA					NA
Coronary	8 (3.56)	0 (0.00)	4 (5.13)	4 (5.41)	0.136	4.27 (1.78)	0.00 (0.00)	2.13 (3.00)	2.15 (2.18)	0.298
Cerebral	23 (10.22)	3 (4.11)	8 (10.26)	12 (16.22)	0.053	20.51 (8.55)	7.59 (10.76)	6.33 (8.93)	6.59 (6.69)	0.767
Visceral	20 (8.89)	3 (4.11)	7 (8.97)	10 (13.51)	0.134	20.83 (8.68)	8.00 (11.33)	6.54 (9.23)	6.29 (6.38)	0.709
Peripheral	19 (8.44)	3 (4.11)	7 (8.97)	9 (12.16)	0.210	15.02 (6.26)	4.23 (5.99)	6.13 (8.66)	4.66 (4.73)	0.658
Extension of dissection					NA					NA
Supra-aortic vessels	32 (14.22)	7 (9.59)	11 (14.10)	14 (18.92)	0.269	24.48 (10.20)	5.88 (8.34)	8.88 (12.54)	9.71 (9.85)	0.747
Iliac vessels	23 (10.22)	3 (4.11)	8 (10.26)	12 (16.22)	0.053	19.66 (8.19)	5.84 (8.28)	6.89 (9.74)	6.92 (7.02)	0.861
Laboratory data										
Leucocytes(10^9^/L)	12.67 ± 3.83	12.69 ± 4.43	12.58 ± 3.16	12.74 ± 3.89	0.968	12.18 ± 3.69	12.37 ± 4.44	12.61 ± 3.11	11.72 ± 3.47	0.543
Hemoglobin (g/L)	130.71 ± 19.19	135.26 ± 18.50	128.64 ± 18.11	128.41 ± 20.38	0.047	134.46 ± 18.41	136.03 ± 18.62	130.18 ± 18.39	136.41 ± 17.96	0.193
Serum creatinine (mmol/l)	112.05 ± 106.26	90.40 ± 28.95	83.91 ± 30.41	163.0 ± 169.97	< 0.001	107.33 ± 84.16	99.96 ± 31.45	86.80 ± 30.75	127.3 ± 123.52	0.019
Albumin (g/L)	37.72 ± 6.72	39.34 ± 6.31	37.84 ± 6.26	36.01 ± 7.23	0.010	39.06 ± 7.72	38.11 ± 5.63	37.94 ± 6.28	40.55 ± 9.58	0.566
Surgical data										
Aortic root procedure					0.003					0.376
No treatment	69 (30.67)	25 (34.25)	22 (28.21)	22 (29.73)		56.53 (23.56)	17.83 (25.26)	20.42 (28.84)	18.28 (18.54)	
Sinus plasty	106 (47.11)	22 (30.14)	42 (53.85)	42 (56.76)		112.77 (46.99)	37.36 (52.94)	34.93 (49.33)	40.48 (41.06)	
Bentall procedure	50 (22.22)	26 (35.62)	14 (17.95)	10 (13.51)		70.67 (29.45)	15.38 (21.80)	15.46 (21.83)	39.83 (40.40)	
Concomitant CABG	8 (3.56)	0 (0.00)	4 (5.13)	4 (5.41)	0.136	3.86 (1.61)	0.00 (0.00)	2.04 (2.88)	1.83 (1.85)	0.259
Operative time (min)	309.20 ± 69.31	309.90 ± 73.96	316.00 ± 70.92	301.30 ± 61.64	0.272	312.59 ± 61.75	309.15 ± 63.77	307.41 ± 67.63	321.87 ± 57.59	0.221
CPB time (min)	148.41 ± 46.90	149.55 ± 52.59	152.88 ± 49.81	142.57 ± 36.70	0.388	151.53 ± 42.45	149.14 ± 44.10	144.97 ± 44.50	157.95 ± 39.17	0.458
ACC time(min)	49.99 ± 24.55	49.26 ± 24.07	50.15 ± 26.23	50.53 ± 23.47	0.950	48.63 ± 21.54	49.74 ± 21.09	48.66 ± 24.74	47.80 ± 19.51	0.860
SACP time (min)	9.20 ± 3.41	9.09 ± 3.46	9.46 ± 3.78	9.04 ± 2.96	0.715	8.72 ± 3.21	9.22 ± 3.66	9.15 ± 3.35	8.06 ± 2.63	0.256
24 h bleeding volume(ml)	496.75 ± 344.74	498.6 ± 357.70	462.8 ± 365.71	530.5 ± 308.25	0.483	481.7 ± 313.37	494.5 ± 318.21	472.9 ± 363.03	478.8 ± 271.76	0.939

Values are given as median and interquartile range or numbers and percentages.

BMI, body mass index; LVEF, left ventricular ejection fraction; CABG, coronary artery bypass grafting; CPB, cardiopulmonary bypass; ACC, aortic cross-clamp; SACP, selective antegrade cerebral perfusion.

### Perioperative outcomes

In-hospital death occurred in 22 patients (2 in Tertiles 1, 5 in Tertiles 2, 15 in Tertiles 3, respectively) and the mortality rate was 9.8% (2.74% vs. 6.41% vs. 20.27%, *p* = 0.001). This difference also remained statistically significant (*p* = 0.024) after IPTW weighting. Before IPTW, A higher incidence of renal failure in patients with a high ACEF score was observed (16.44% vs. 24.36% vs. 41.89%, *p* = 0.002), however this effect was no longer found after weighting (*p* = 0.777). There was a statistically significant difference in hepatic insufficiency among groups (*p* = 0.035) after IPTW, while it was not apparent before IPTW. No significant differences were observed in the rest complications after IPTW. The comparison of perioperative outcomes between the groups before and after matching is summarized in Table 2. In ROC curve analysis, the discriminative capacity of the ACEF score was better than ACEF II score in predicting in-hospital mortality (Fig. 4a). The AUC of ACEF, ACEF II score was 0.723 (95% CI 0.613–0.833; *p* = 0.006), 0.642 (95% CI 0.512–0.771; *p* = 0.029), respectively. But there is no significant difference (*p* = 0.127).

**Table 2:** Comparisons of perioperative outcomes according to ACEF score groups after before matching and after IPTW.

Characteristics	ACEF score tertiles									
	Before matching					After IPTW				
	Overall	≤ 0.73	0.73–0.95	> 0.95	*p*	Overall	≤ 0.73	0.73–0.95	> 0.95	*p*
n	225	73	78	74	NA	239.97	70.57	70.81	98.6	NA
In-hospital mortality	22 (9.78)	2 (2.74)	5 (6.41)	15 (20.27)	0.001	22.44 (9.35)	2.03 (2.88)	3.27 (4.62)	17.13 (17.38)	0.024
Permanent neurological deficits	43 (19.11)	13 (17.81)	11 (14.10)	19 (25.68)	0.182	36.84(15.35)	17.79 (25.21)	8.07 (11.39)	10.99 (11.15)	0.106
Low cardiac output syndrome	33 (14.67)	6 (8.22)	11 (14.10)	16 (21.62)	0.070	27.41 (11.42)	9.81 (13.90)	8.51 (12.02)	9.08 (9.21)	0.764
Cardiogenic shock	4 (1.78)	0 (0.00)	3 (3.85)	1 (1.35)	0.191	2.35 (0.98)	0.00 (0.00)	2.02 (2.85)	0.33 (0.34)	0.054
Secondary intubation	8 (3.56)	0 (0.00)	3 (3.85)	5 (6.76)	0.085	7.41 (3.09)	0.00 (0.00)	2.07 (2.93)	5.34 (5.41)	0.244
Tracheotomy	17 (7.56)	3 (4.11)	8 (10.26)	6 (8.11)	0.352	19.82 (8.26)	8.87 (12.57)	6.90 (9.74)	4.06 (4.11)	0.359
Renal failure	62 (27.56)	12 (16.44)	19 (24.36)	31 (41.89)	0.002	63.93 (26.64)	17.61 (24.95)	16.43 (23.20)	29.89 (30.32)	0.777
Hepatic insufficiency	56 (24.89)	12 (16.44)	19 (24.36)	25 (33.78)	0.052	85.50 (35.63)	16.97 (24.04)	15.45 (21.82)	53.09 (53.84)	0.035
Sepsis	27 (12.00)	4 (5.48)	11 (14.10)	12 (16.22)	0.105	33.57 (13.99)	8.80 (12.47)	9.31 (13.15)	15.46 (15.68)	0.923
Gastrointestinal bleeding	29 (12.89)	6 (8.22)	9 (11.54)	14 (18.92)	0.139	36.02 (15.01)	10.58 (14.99)	6.82 (9.62)	18.63 (18.90)	0.598
Re-operation	1 (0.44)	1 (1.37)	0 (0.00)	0 (0.00)	0.351	0.53 (0.22)	0.53 (0.75)	0.00 (0.00)	0.00 (0.00)	0.657
Sternal wound infection	11 (4.89)	2 (2.74)	3 (3.85)	6 (8.11)	0.278	16.22 (6.76)	1.46 (2.07)	2.88 (4.07)	11.87 (12.04)	0.126

Values are given as numbers or numbers and percentages.

In-hospital mortality, death within the same hospital admission or death from any-cause 30-day post-procedure.

Permanent neurological deficits, stroke with positive neuroimaging findings.

Low cardiac output syndrome, patients requiring intra-aortic balloon pump insertion.

Cardiogenic shock, persistent hypotension (systolic blood pressure < 90 mmHg) with the cardiac index (CI) < 1.8 L/(min·m^2^).

Secondary intubation, patients requiring re-intubation because of respiratory failure.

Tracheotomy, patients requiring tracheostomy for long-term ventilator dependence.

Renal failure, serum creatinine increased by > 3 times the baseline values, GFR decreased by > 75%, oliguria: urine output < 0.3 mL·kg^−1^·h^−1^ for 24 h, or anuria > 12 h or requiring temporary hemodialysis support for resolution.

Hepatic insufficiency, hepatobiliary ischemia manifested as metabolic acidosis or increased lactate or prothrombin time, requiring general surgeon consultation.

Sepsis, a severe infection accompanied by symptoms and signs of a primary infectious focus, and positive blood culture results for bacteria.

Gastrointestinal bleeding, patients with evidence of gastrointestinal bleeding, accompanied by a significant progressive decrease in hemoglobin.

Re-operation, patients requiring re-operation on in the immediate postoperative period because of bleeding.

Sternal wound infection, deep wound infection involving the sternum, requiring surgical intervention under general anesthesia, or wound dehiscence requiring reapproximation of the sternum under general anesthesia.

### 1-year outcomes

**Figure 3:**
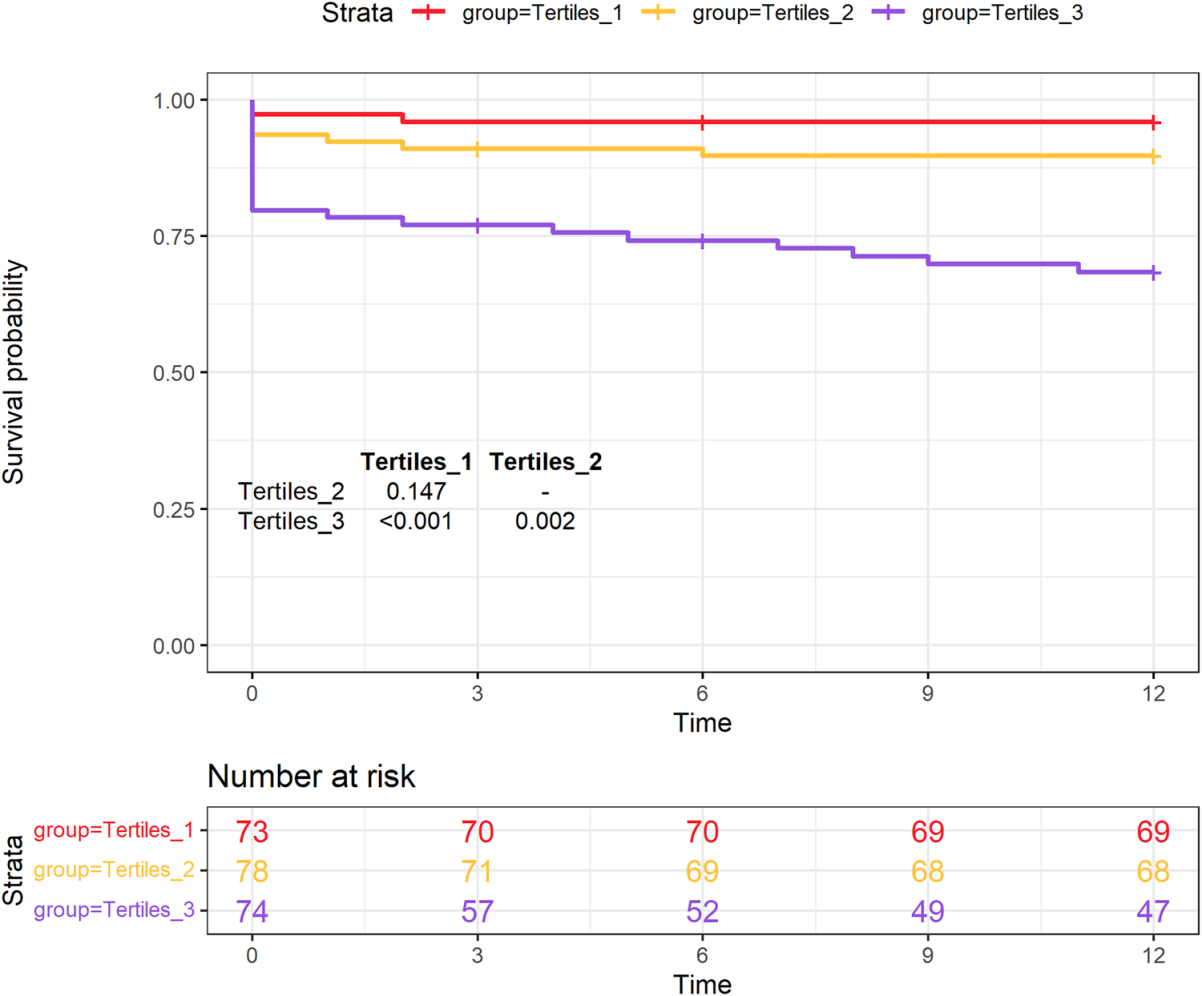
Kaplan–Meier curves of 1-year survival in patients with different ACEF groups.

**Figure 4:**
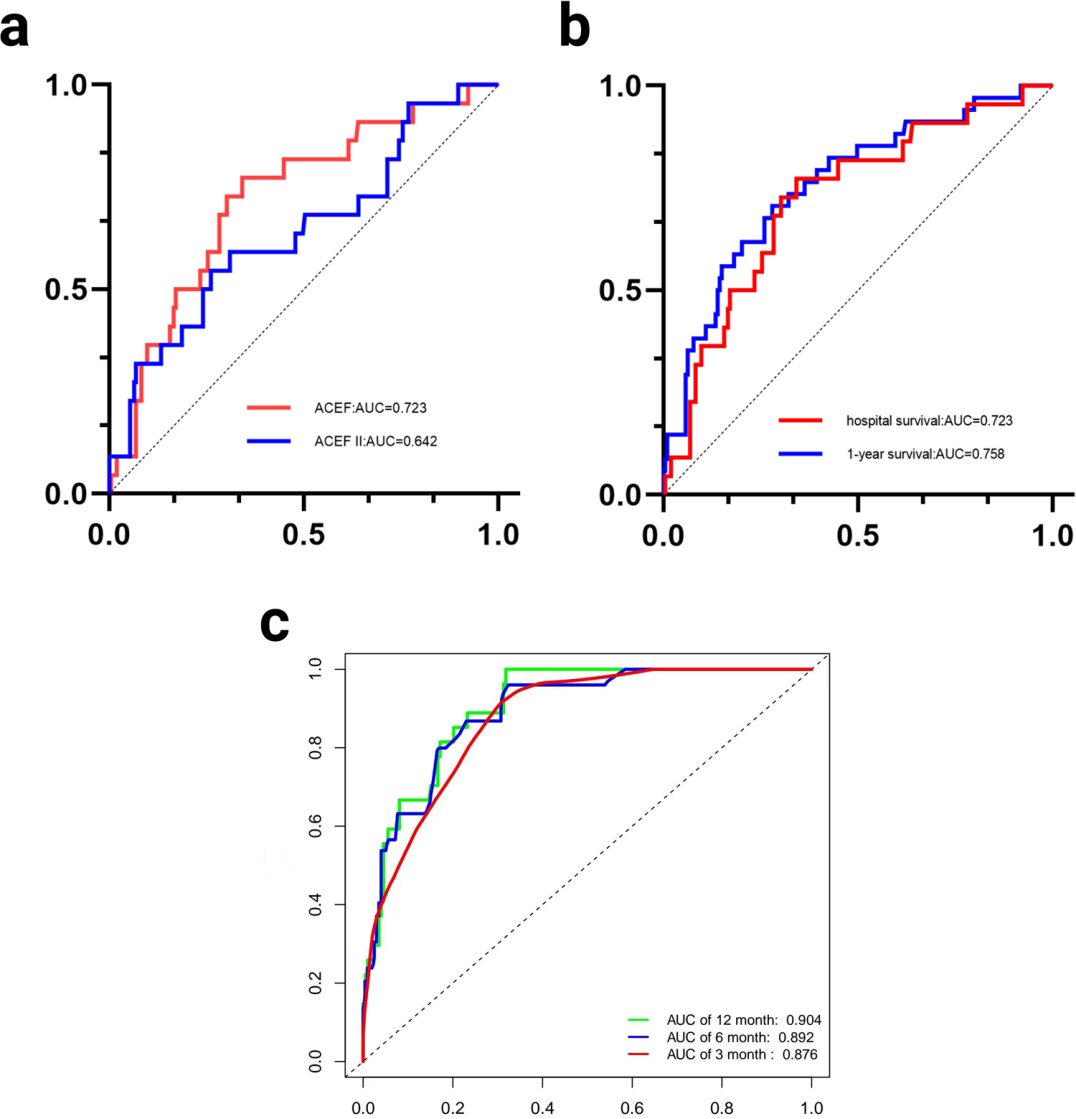
ROC curves of ACEF score and ACEF IIin predicting in-hospital (**a**) ROC curves of ACEF score in predicting in-hospital at different time (**b**) Time-dependent ROC curve of binary ACEF model (**c**).

All 203 patients discharged completed the 1-year follow-up. 1-year survival was 90.6% (97.18% vs. 93.15% vs. 79.66%, respectively). The results showed that the baseline ACEF score were significantly affected 1-year survival outcomes (log-rank test, *p* (Tertiles 1:2) = 0.147, *p* (Tertiles 1:3) < 0.001, *p* (Tertiles 2:3) = 0.002). Figure 3 shows the survival curves of Tertiles 1, 2, and 3 by Kaplan–Meier methods. The results of univariable and multivariable Cox proportional hazard analyses are presented in Table 3. To maximize the predictive value, the receiver operating characteristic (ROC) analysis was performed and cut-off values of ACEF in 0.94 (sensitivity, 0.706; specificity, 0.717; AUC, 0.758) were obtained (Fig. 4b). In the univariate analysis, the ACEF [hazard ratio (HR) 2.98; 95% CI 1.06–8.36; *p* = 0.038] and ACEF > 0.94 [HR 4.95; 95% CI 2.36–10.36; *p* < 0.001] were a significant predictor of 1-year all-cause mortality. However, ACEF II was not an independent risk factor for death. After adjusting for potential risk factors (BMI, cerebral malperfusion, dissection involving supra-aortic vessels), ACEF (adjusted hazard ratio = 1.68; 95% CI 1.34–4.91; *p* = 0.036) and ACEF > 0.94 (adjusted hazard ratio = 2.26; 95% CI 1.82–6.20; *p* < 0.001) remained an independent predictor for 1-year mortality.

**Table 3:** Cox regression analyses for 1-year outcomes according to ACEF score groups.

Parameters	Univariate analysis		Multivariate analysis	
	HR (95% CI)	*P*	HR (95% CI)	*P*
Male	1.09 (0.45–2.69)	0.845		
BMI	0.79 (0.69–0.92)	0.002	0.88 (0.78–0.98)	0.025
Resuscitation before surgery	1.82 (0.20–16.45)	0.594		
Emergency surgery	2.14 (0.32–4.54)	0.967		
Inotropes at referral	2.77 (0.56–6.78)	0.210		
Hemiparesis	1.39 (0.29–1.94)	0.683		
Preoperative ventilation	2.60 (0.58–11.59)	0.191		
Previous cardiac surgery	3.11 (0.31–30.99)	0.333		
Aortic valve regurgitation	1.50 (0.34–6.67)	0.152		
Pericardial effusion	2.86 (0.47–8.99)	0.900		
Coronary malperfusion	1.95 (0.28–13.78)	0.504		
Cerebral malperfusion	2.90 (1.15–7.29)	0.023	2.52 (1.18–5.40)	0.017
Visceral malperfusion	2.51 (0.71–8.87)	0.152		
Dissection involving supra–aortic vessels	5.58 (2.04–15.31)	< 0.001	5.58 (2.58–12.05)	0.008
Dissection involving iliac vessels	3.00 (1.11–8.11)	0.031	2.11 (0.91–4.91)	0.084
Albumin	1.05 (0.98–1.12)	0.141		
Leucocytes	1.10 (0.98–1.23)	0.103		
ACEF	2.98 (1.06–8.36)	0.038	1.68 (1.34–4.91)	0.036
ACEF > 0.94	4.95 (2.36–10.36)	< 0.001	2.26 (1.82–6.20)	< 0.001
ACEF II	1.12 (0.95–1.14)	0.127		
Concomitant CABG	2.37 (0.22–24.98)	0.474		
CPB time	1.01 (0.99–1.01)	0.308		
ACC time	1.01 (0.98–1.02)	0.940		

Values are given as numbers or numbers and percentages.

CABG, coronary artery bypass grafting; CPB, cardiopulmonary bypass; ACC, aortic cross-clamp.

### Clinical application of ACEF score

A prediction model was constructed from baseline factors including BMI, cerebral malperfusion, dissection involving supra-aortic vessels, the addition of continuous ACEF score or binary ACEF score (transformed to a categorical variable based on the cut-off value generated by ROC analysis) improve Harrell’s C-index for 1-year mortality (0.877 and 0.879). However, the time dependent NRI and IDI tended both to be improved in binary ACEF score model (0.435, *p* < 0.001, and 0.131, *p* < 0.001), respectively (Table 4). The time-dependent AUC (Fig. 4c) shows that the AUC of binary ACEF score model continues to increase within 1 year postoperatively (3, 6, 12 month: 0.876, 0.892, 0.904).

**Table 4:** Predictive accuracy of the ACEF score using Harrell’s C-index for Cox hazard model, time dependent NRI and time dependent IDI.

Parameters	C-index	*P*	Time dependent NRI	*P*	Time dependent IDI	*P*
Baseline risk factors	0.814					
+ ACEF (continuous)	0.877	0.041	0.413	< 0.001	0.060	0.182
+ ACEF > 0.94	0.879	0.033	0.435	< 0.001	0.131	< 0.001

Values are given as numbers or numbers and percentages.

NRI, net reclassification improvement; IDI, integrated discrimination improvement.

## Discussion

The current investigation showed that the predictive value of the ACEF score in early surgical outcome in AAAD patients underwent total arch replacement. Patients in a higher ACEF score possibly indicating a poor baseline characteristic, with more comorbidities and prior significant medical history. This lack of consistency made direct comparison difficult between groups. Thus, we applying the IPTW to balance the baseline and surgical variables without reducing the sample size, making the conclusion more persuasive and scientific. The key advantage of ACEF score is its simplicity and rapidity, no specific software is necessary. The ACEF score could be considered as a useful tool to risk stratification in patients with AAAD before operation in daily clinical work.

Acute type A aortic dissection (AAAD) is a life-threatening pathology. The mortality is reportedly 9.4–22%^1,16^.Despite the advances in surgical techniques and perioperative care, the short-term outcome is still undesirable with in-hospital mortality at 9.8% and 1-year survival at 90.6%. Previous studies suggested that the AAAD surgical outcome was closely related to preoperative state^17^. However, a comprehensive evaluation within the limited time is admittedly a challenging task. Therefore, it is necessary to find a rapid and easy method to identify high-risk patients. Then, a comprehensive baseline data evaluation of high-risk patients should be taken to developing an optimize treatment strategies. Currently, ACEF score has been considered as a useful risk stratification tool in many cardiovascular disease patients^18–20^. The advanced age, high level of serum creatinine and the worse cardiac function has also been acknowledged as the important independent predictive factor for AAAD patients^21–23^. In our study, by comparing AAAD patients in different ACEF score, although the baseline has been balanced, a higher ACEF score was strongly associated with a higher in-hospital mortality. In all discharged patients, the 1-year survival still affected by a low baseline ACEF score.

The ACEF score, comprising only three key variables, is undoubtedly not a substitute for well-recognized scores such as the EuroSCORE II and the GERAADA score in the assessment model for Stanford type A acute aortic dissection. It cannot accurately calculate surgical mortality. Its significance lies particularly in the quick and effective identification of high-risk patients. This provides a reference for optimizing clinical decision-making in high-risk patients, including further comprehensive assessment, more aggressive use of support devices, simplified surgical procedures, and close postoperative monitoring. In the meantime, ACEF score also has a certain predictive role in the 1-year outcomes of AAAD patients. For patients in high ACEF score, intensive follow-up, and guidance in early postoperative are necessary.

The ACEF II score, a modified version of the ACEF score that includes the variables emergency surgery and preoperative anemia, is already being widely used^24^. There is no doubt that emergency surgery and preoperative anemia are both risk factors of thoracic aortic surgery. In the current study, however, ACEF II score did not show a good prediction ability for in-hospital mortality and 1-year survival in AAAD patients underwent total arch replacement. One reason for this might be the vast majority of the AAAD patients (88.00%) underwent emergency surgery and the assignment value of emergency surgery in ACEF II score was much high (3 (if emergency surgery)). It may lead to the overestimation of actual mortality rate. Hence, for better prediction of mortality in AAAD patients, the equations of ACEF II scores should be recalibrated.

It is strange that renal failure is no more a significant variable between groups after weighing the baseline and surgical variables. In the current study, concomitant chronic renal disease is the main cause of the preoperative elevated creatinine level. We can only speculate that postoperative acute renal failure is considerably more often caused by acute ischemia.

## Limitation

Several limitations of this study should be considered. On top of all of that, the retrospective nature of this study introduces inherent biases, causality cannot be determined. Next, this study was conducted in a single center, the limited sample size and a short follow-up duration may influence the extrapolation to all institutions. Thus, the multicenter studies with a large sample size and long follow-up period are warranted to better replicate our results. Also important is, despite the comprehensive IPTW weighting, residual confounding might remain.

## Conclusions

In this study, the ACEF score, was demonstrated to be associated with in-hospital mortality and 1-year survival of AAAD patients after total arch replacement. As a simple and reliable score, ACEF could be considered as a useful tool to risk stratification in patients with AAAD before operation in daily clinical work.

## Data Availability

The data that support the findings of this study are available on request from the corresponding author.

## Abbreviations

ACEF: Age, creatinine, and ejection fraction
AAAD: Acute type A aortic dissection
IPTW: Inverse probability processing weighting
NRI: Net reclassification improvement
IDI: Integrated differentiation improvement
EuroSCORE II: European System for Cardiac Operative Risk Evaluation II
GERAADA: German Registry for Acute Aortic Dissection Type A score
CTA: Computed tomography angiography
ROC: Receiver-operating characteristic curve
AUC: Areas under the curve
